# Frequency Response Stabilization and Comparative Studies of MET Hydrophone at Marine Seismic Exploration Systems †

**DOI:** 10.3390/s20071944

**Published:** 2020-03-30

**Authors:** Egor Egorov, Anna Shabalina, Dmitry Zaitsev, Sergey Kurkov, Nikolay Gueorguiev

**Affiliations:** 1 Department of Physical and Quantum Electronics, Moscow Institute of Physics and Technology, Dolgoprudny 141700, Russia; shabalina@phystech.edu (A.S.); zaitcev.dl@mipt.ru (D.Z.); kurkov.sv@mipt.ru (S.K.); 2 LLC R-Sensors, R&D department, Dolgoprudny 141700, Russia; 3 Department of Mechanical Devices, Institute of Metal Science, 1574 Sofia, Bulgaria; niki0611@abv.bg

**Keywords:** hydrophone, acoustic sensor, molecular-electronic technology, negative electrodynamic feedback, seismic exploration, geoscience

## Abstract

Low frequency hydrophone with a frequency range of 1−300 Hz for marine seismic exploration systems has been developed. The operation principle of the hydrophone bases on the molecular electronic transfer that allows high sensitivity and low level self-noise at low frequencies (<10 Hz) to be achieved. The paper presents a stabilization method of the frequency response within the frequency range at a depth up to 30 m. Laboratory and marine tests confirmed the stated characteristics as well as the possibility of using this sensor in bottom marine seismic systems. An experimental sample of the hydrophone successfully passed a comparative marine test at Gelendzhik Bay (Black Sea) with the technical support of Joint-Stock Company (JSC) “Yuzhmorgeologiya”. One of the main results is the possibility of obtaining high-quality information in the field of low frequencies, which was demonstrated in the course of field tests.

## 1. Introduction

Inertial motion sensors are the main elements of all mineral exploration systems. Currently, in addition to widely used electromechanical geophones, electrochemical geophones [1,2] operated on the principles of molecular-electronic transfer (MET) [3,4], have begun to be used in such land systems [5,6].

MET technology allows for the design of highly sensitive sensors operating in a low frequency range—lower than 0.1 Hz. The developed devices are already widely used in seismology [7,8], building monitoring [9,10,11], navigation [12], motion control [13], and healthcare applications [14].

The advantages of MET technology for the sensing industry in different fields are the low production cost with worthy output parameters and high suitability for mass production. All this makes it interesting to search for technical solutions based on MET for various areas of sensor instrumentation including systems for the marine seismic exploration of minerals.

Hydrophones are one of the main components of marine seismic exploration systems. In previous works [15,16], a model of a MET hydrophone was presented. An experimental sample of the hydrophone was also made and laboratory studies of its main characteristics such as amplitude–frequency response and self-noise were carried out [17,18].

Most marine explorations to search for mineral deposits are undertaken in shelf, coastal, and transition zones, where the depth does not exceed 100 m. Laboratory tests have shown that in order to stabilize the amplitude–frequency characteristic, it is necessary to increase the rigidity of the air bubble in the upper cover. The developed theoretical model has shown that in order to do that, it is necessary to use a fluid with a constant isothermal modulus of elasticity. For this purpose, it was decided to fill this volume with compressing fluid, and polymethylsiloxane liquid was chosen.

This work is a detailed and expanded study, presented in [19], demonstrating the development of a highly sensitive molecular electronic (MET) hydrophone with a wide frequency range (1−300 Hz), which maintains stable performance up to a depth of 30 m. As a result, the hydrophone design was refined and laboratory tests of its parameters were carried out. Comparative tests of the MET hydrophone and standard piezoelectric hydrophone in marine conditions were conducted.

## 2. Stabilization Method of the Frequency Response of the Hydrophone

The detailed scheme and principle of operation of the MET transducer element and hydrophone (METH) were described in the papers [3,17].

The transfer function of METH consists of two subsystems: the mechanical one with the W_mech_ transfer function and the electrochemical one with W_elch_ transfer function.

The mechanical subsystem denotes the transfer of the external pressure variations to the electrolyte volumetric flow, whereas the electrochemical part transfers the electrolyte inner flow to the signal current of METH.

**Figure 1:**
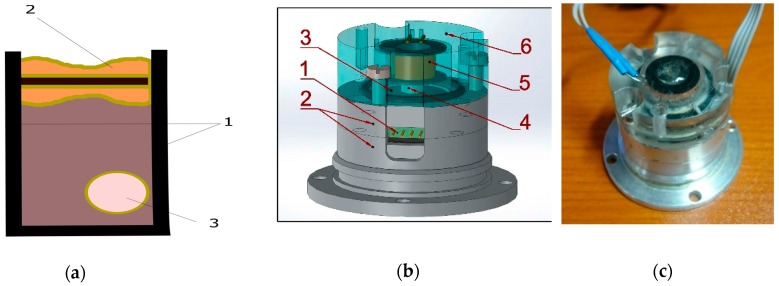
Hydrophone: (**a**) scheme; (**b**) design [19]; (**c**) experimental sample [19].

The schematic structure of the mechanical system of the MET hydrophone is shown in Figure 1a. It consists of durable non-deformable housing (1), transducing element in volume filled with working fluid and limited by membranes (2), and air bubbles in silicon fluid (3). One of its membranes is in contact with the external medium, where the pressure variations occur. The second membrane is in contact with the volume inside the hydrophone housing filled with compressing fluid. The principle of operation is that the external pressure acts on the external membrane and drives the liquid in the transducing element due to the compressibility of the liquid filling the internal volume of the hydrophone housing. The volumetric flow q of the working fluid through the sensing element can be represented as follows [17]:

(1) $$q=\frac{P_{ex}e^{i\omega t}-P_{in}-\frac{\rho \dot{q}l}{s}}{R_{h}},$$

where P_ex_ denotes the amplitude of external pressure changing with frequency ω; P_in_ denotes the pressure in the internal volume of the hydrophone housing; s denotes the hydrophone cross-sectional area; l denotes the hydrophone length; and R_h_ denotes the hydrodynamic resistance to fluid flow through the sensing element.

If the internal pressure is represented as $P_{in}=\frac{\partial P}{\partial V}\Delta V=\frac{\partial P}{\partial V}\int^{\;}qdt$, then the complex amplitude of the harmonic solution of the equation can be represented as follows:

(2) $$q=\frac{P_{\mathrm{ex}}}{R_{h}\left(1+\frac{\partial P}{\partial V}\frac{1}{{i\omega R}_{h}}+\frac{\rho l}{R_{h}s}i\omega \right)},$$

and the mechanical part of the METH whole transfer function will be next:

(3) $$W_{\mathrm{mech}}=\left|\frac{q}{\mathrm{Pex}}\right|.$$

**Figure 2:**
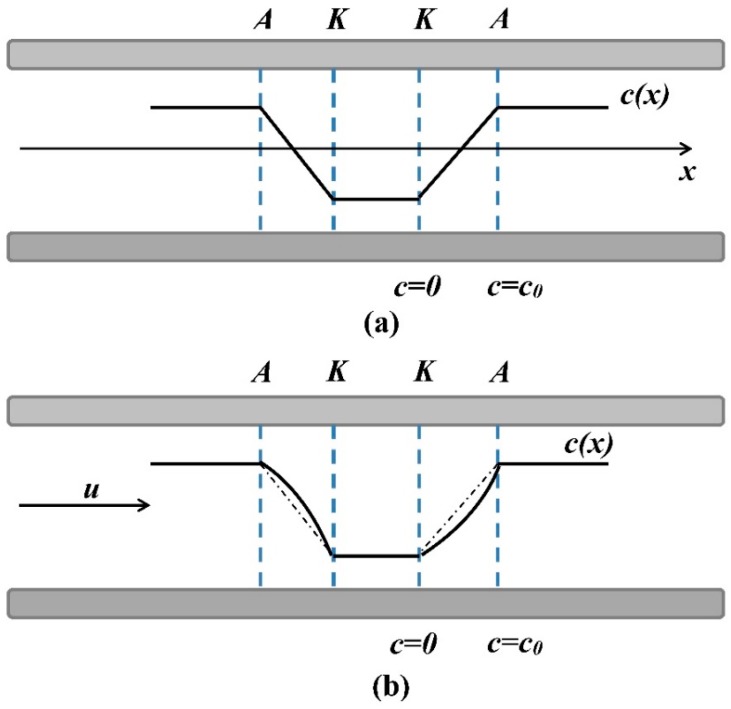
The carrier concentration gradient in the transducing cell under the influence of external pressure variations. c(x) is the ion concentration; (**a**) distribution without external pressure variations; (**b**) the distribution of concentration c(x) varies under the oncoming flow of liquid; u = fluid flow rate. A = anode, K = cathode.

Regarding the electrochemical part of the transducing process, it is the same as in all types of molecular electronics devices [20], and the whole transducing mechanism can be clarified from Figure 2. The transducing cell (four electrodes anode (A)–cathode (K) pairs) is immersed in liquid electrolyte. A small voltage is applied to the anodes in relation to the cathodes (~300 mV). An oxidation–reduction reaction on the electrodes occurs. At the anodes, an excess number of current carriers is formed; at the cathodes, these carriers recombine. As a result, a gradient of the concentration of current carriers is formed between each pair of anode–cathode, which changes when fluid starts to flow due to external pressure variations.

The transfer function of the electrochemical system is described by Navier–Stokes equations, non-compressibility of the liquid, and convective diffusion, as described in [20]. For a known concentration distribution, the cathode currents can be found as:

(4) $$I=-De\oint_{S}\left(\nabla c,\vec{n}\right)dS.$$

Here, integration is performed over the electrode surface S; $\vec{n}$ is a unit vector normal to the surface; e is the charge transferred through the electrode in a unit reaction; D is the diffusion coefficient; and c is the active electrolyte ion concentration.

(5) $$W_{el-ch}=\frac{ec_{0}}{{\left(1+\frac{\omega^{2}}{\omega_{el-ch}^{2}}\right)}^{1/2}},$$

where $\omega_{el-ch}=bD/d^{2}$; d is the distance between the electrodes; b is the parameter depending on the electrode system geometry; and $c_{0}$ is the equilibrium concentration.

The MET accelerometer with negative electrodynamic feedback was taken as the basis of the hydrophone (Figure 1). The sensing element (4-electrode cell (1)) was placed in the volume between the metal flanges (2). Elastic rubber membranes (3) limit the volume from both sides. The elasticity of the membranes allows the electrolyte to move through the sensing element. To form the feedback, a magnet (4) is attached to one of the membranes, and an electromagnetic coil (5) is fixed in the upper cover (6). The geometrical parameters of the cover and the coil are selected in such a way that the magnet can move freely, according to the influence of the Lorentz force. With open membranes, the electrochemical system registers the movement of the surface on which the accelerometer is installed. While one membrane is hermetically closed with the cover, the system begins to feel a pressure change via the open membrane.

If there is an air bubble under the cover during assembly, it is at the pressure of 1 atm. In this case, if the constant pressure on the outer membrane increases (for example, when the hydrophone is immersed), the inner membrane will flex until the pressure levels are balanced.

It is obvious that the volume V of the internal bubble will decrease inversely to the external pressure, which is equal to the pressure P inside the air bubble. First, this leads to the fact that the inner membrane will tighten against the coil and thereby distort the output signal.

Second, the rigidity of the system, which equals dP/dV increases. Taking into account for the volume V of the bubble under the cover, P_0_V_0_ = P_1_V_1_, where P_0_ = 1 atm., V_0_ is the initial bubble volume at the pressure of 1 atm., V_1_ is the bubble volume at the pressure P_1_; in our case, the rigidity of the system is determined by the following equation:

dP/dV = P/V = P^2^/(P_0_V_0_). (6)

However, by increasing the external pressure, the increase in rigidity leads to the decrease in sensitivity at high frequencies. It should also be noted that the feedback mechanism cannot compensate the constant pressure.

To fix one of the issues, we proposed filling the volume with compressible liquid.

The compressibility of liquids is characterized by compressibility factor k = V^−1^dV/dP or its reciprocal E = V dP/dV, which is the isothermal modulus of elasticity.

Of all the liquids common in engineering, silicone fluids are the most compressible. This fluid is chemically inert, corrosion-resistant, and has good dielectric and damping properties. It has two times less volumetric elastic modulus (~1030 MPa) than water (~2060 MPa).

Equation (6) and the METH design in Figure 1 imply that at shallow depths, METH with the volume filled with air has less rigidity and, accordingly, higher sensitivity. However, the rigidity of the air bubble greatly increases with depth, in contrast to the rigidity of the silicone. Thus, for a deep-water hydrophone (up to 100 m), the use of silicone fluid may be a reasonable solution. This is overall more significant because the total deformation of the membranes of the sensing element in the depth will be less, which means that the sensitivity of the hydrophone is less dependent on the immersion depth.

It is worth pointing out that the frequency range of the mechanical subsystem is not directly related to the frequency range of the designed sensor if negative feedback is used. The functioning of the sensor in the feedback loop is discussed in [21]. The frequency range and the sensitivity are more related to the hydrodynamic resistance of the electrode cell of the sensing element [21], and to a lesser extent affects the frequency response stability.

## 3. Laboratory Tests

### 3.1. Frequency Response Characteristic Measurement

**Figure 3:**
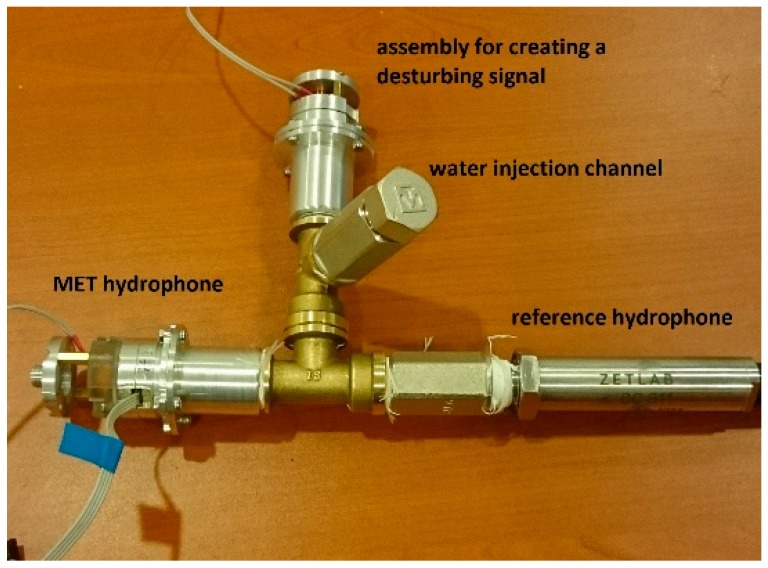
The hydraulic bench.

Two experimental model MET hydrophones were assembled. Their sensitivity was adjusted to 0.8 mV/Pa ± 1 dB in the range of 1−300 Hz (−3 dB at the boundaries). The isolated volume of one of them was completely filled with polymethylsiloxane liquid. The isolated volume of the second one was partially filled with the same liquid and with air. Tests of its frequency response in the range of pressure differences up to 3 atm. were carried out on a special hydraulic bench (Figure 3).

The hydraulic bench consists of a system of pipes that connects the MET hydrophone, reference hydrophone, and solenoid assembly to input a disturbing signal. Water was pumped through the water injection channel under a certain pressure. A sinusoidal signal current was supplied to the solenoid in the required frequency range. As a result of comparing the spectra of the hydrophone signals, the frequency response characteristic of the MET hydrophone was obtained. More details about obtaining the frequency response characteristic are described in [17].

**Figure 4:**
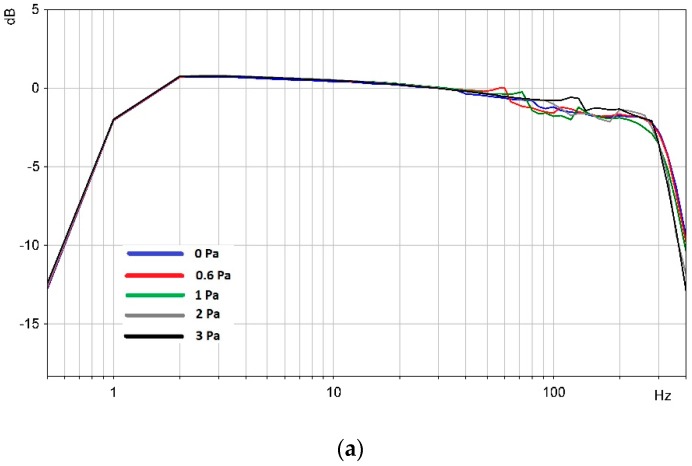

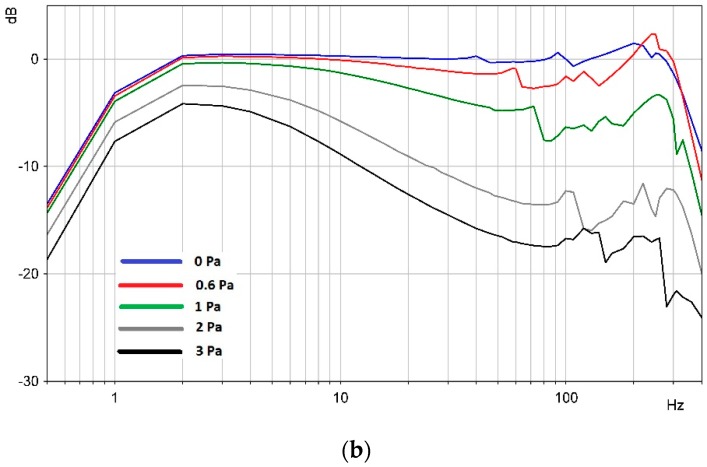
Frequency response curves (0.8 mV/Pa) of the MET hydrophones under different pressure differences. (**a**) With the volume completely filled with liquid under the upper cover; (**b**) with the partially filled volume.

Calibration results are shown in Figure 4.

Laboratory tests showed that the frequency response of the MET hydrophone with a completely filled volume under the upper cover varied by −0.1 dB (~1%) at low frequencies and by −0.5 dB (~6%) at high frequencies with increasing pressure difference on the membranes to 3 atm.

Similar tests with a hydrophone with a partially filled volume of liquid showed that the frequency response curve was more unstable with increasing pressure differences across the membranes. In this case, the frequency response curve decreased by −4 dB (twice) at low frequencies and by −22 dB (12 times) at high frequencies.

Further tests were carried out with the first type of the MET hydrophone.

### 3.2. Self-Noise Measurement

Self-noise level is also the one of the main characteristics of a hydrophone. Theoretical models were built and studied in [22,23,24]. In these works, the effects of convection processes, cell geometry, and hydrodynamic resistance on the value of the hydrophone’s self-noise were considered.

**Figure 5:**
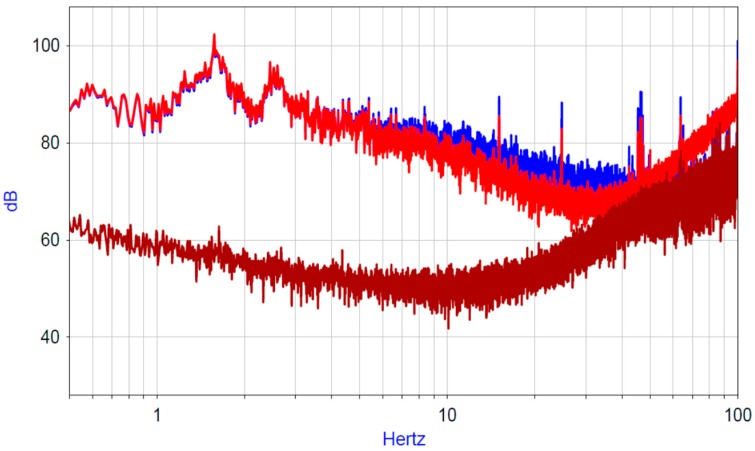
Power spectral density of the hydrophone signals. Blue and light-red lines are the spectra of the hydrophone signals. Red line is the noise spectral density.

In the present study, the hydrophone’s self-noise was measured. For this, two hydrophones were connected coaxially and placed in an isolated pool, which was located in a room isolated from external seismic influences. For the necessary analysis of noise characteristics, the signal of the tested sensor was recorded during a long time period (~10 h) in the absence of external influences and at a constant temperature T = 20 °C. For recording, a high-precision 24-bit data acquisition system NDAS 8662 (Limited Liability Company (LLC) R-Sensors [25]) was used. To determine the device’s self-noise, the signal correlation method was used [21]. Figure 5 shows the measurement results.

Compared with the experimental results in [17,22], it can be seen that the measured self-noise in the presented frequency range was not inferior to that measured for a hydrophone with an air bubble.

## 4. Marine Experiments

**Figure 6:**
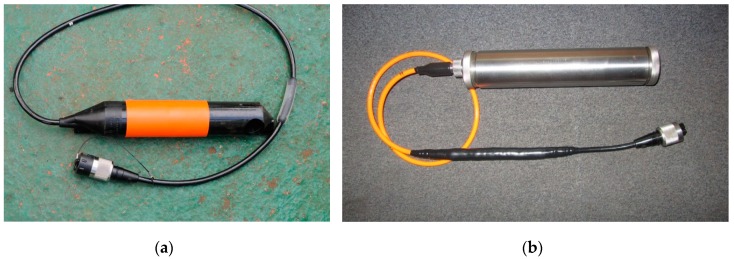
Hydrophones. (**a**) Reference hydrophone at GS-PV-1S; (**b**) molecular-electronic transfer (MET) hydrophone.

Marine tests were held at Gelendzhik Bay (the Black Sea) with technical support from JSC “Yuzhmorgeologiya”. One goal of the tests was to compare the response of the reference hydrophone (Figure 6a) and the MET hydrophone (Figure 6b) to an external disturbing signal generated by a single pneumatic source of 40 cubic inches. Hydrophone MP-25-250, which is a part of the dual sensor GS-PV-1S in the ARAM ARIES II system, was used as the reference hydrophone. It has a sensitivity of 10.2 V/bar in the frequency range from 10 Hz.

**Figure 7:**
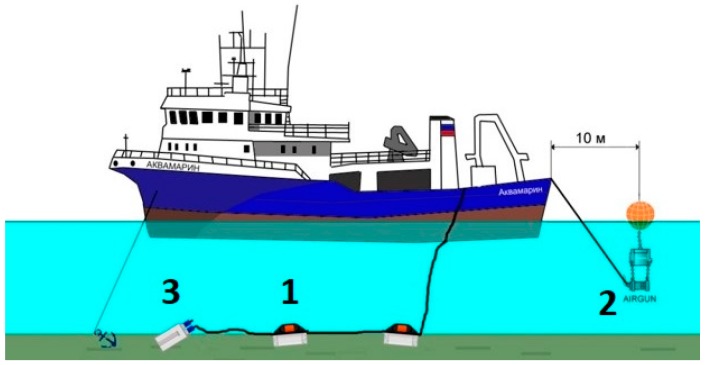
The scheme of the marine test. (**1**) hydrophones to compare; (**2**) single pneumatic source; (**3**) remote acquisition module.

**Figure 8:**
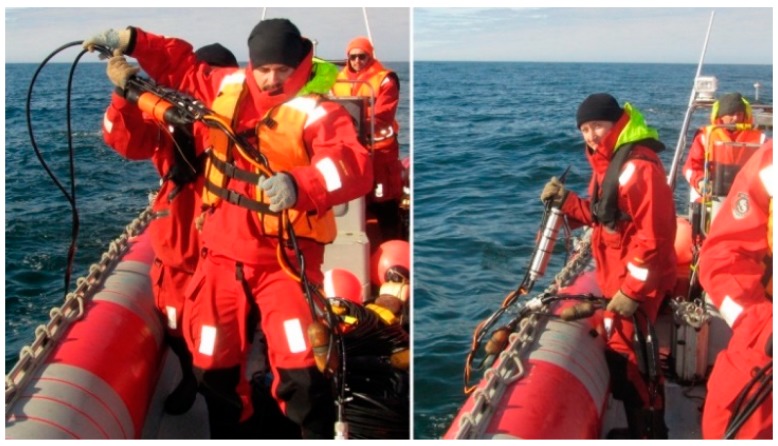
Hydrophone submergence.

**Figure 9:**
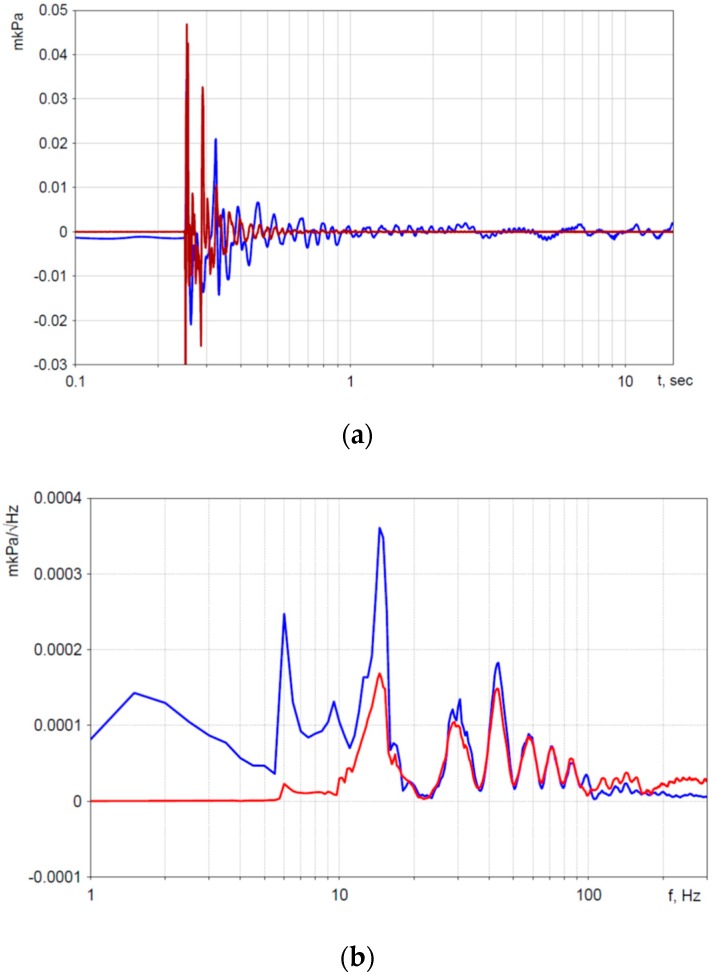
Signal (**a**) and spectra (**b**) of the hydrophone signals of the same shot in the marine test: blue line = MET hydrophone; red line = reference hydrophone.

The scheme of the experiment is shown in Figure 7. The hydrophones were rigidly connected to each other so that their sensitive elements were located at the same point with respect to the shot point at the depth of 15 m (Figure 8). They were connected to a remote acquisition module synchronized with the excitation system. The distance to the source of disturbing signal was about 50 m. The signal recording time was 30 s, with a sampling frequency of 2000 Hz. The spectrum was calculated for the 2 s recording (Figure 9a).

A comparison of two hydrophones was carried out according to the signal spectrum. The spectra of one of the acoustic signals are shown in Figure 9.

There was a good match (up to 20%) of signals in the range from 15 to 100 Hz. The discrepancy at low (1–15 Hz) frequencies was caused by the flat characteristic of the MET hydrophone in this range and the lower cut-off frequency by 10 Hz (–3 dB) at the reference hydrophone. The discrepancy in the range from 100 to 200 Hz could be caused by the inaccuracy of placing the hydrophones at one-point relative to the signal source. The discrepancy in the spectra at high frequencies (from 200 Hz) was caused by the fact that the upper cut-off frequency of the MET hydrophone was 300 Hz, while the frequency response curve of the reference hydrophone at 300 Hz remained flat. The key advantage of a new METH is a high sensitivity at low frequencies (<10 Hz), where one can find new and quality information necessary for seismic exploration, which is not achievable with the use of standard measuring tools.

## 5. Discussion

Laboratory experiments have shown that the proposed method of stabilizing the frequency response acts at depths of up to 30 m. A flat response characteristic is maintained within the stated operating frequency range (from 1 Hz up to 300 Hz). The MET hydrophone’ s self-noise is consistent with previously developed theoretical models [18,21].

Comparing the experimental sample of the MET hydrophone with one of the frequently used hydrophones for marine seismic exploration showed the identity of the information received.

Based on the results obtained, the MET hydrophone can be used in marine seismic exploration systems.

For the final launch of the developed hydrophone to the market, it is necessary to conduct a number of tests and improvements. These include the refinement of the device design and conducting full-fledged seismic exploration tests to obtain the final information in the form of the geological structure of the seabed.

## 6. Conclusions

As a result, the method of stabilizing the frequency response of the MET hydrophone in the range between 1−300 Hz was proposed, tested, and confirmed by laboratory and marine tests. Proven working depths were up to 30 m.

Comparative marine tests of the developed model of the MET hydrophone were carried out, which showed the possibility of using this type of hydrophone in marine seismic exploration systems.

## 7. Patents

Molecular electronic hydrophone RU 2 678 503 C1.
